# Quercetin liposomes protect against radiation-induced pulmonary injury in a murine model

**DOI:** 10.3892/ol.2013.1365

**Published:** 2013-05-29

**Authors:** HAO LIU, JIAN-XING XUE, XING LI, RUI AO, YOU LU

**Affiliations:** 1 Department of Thoracic Oncology, Cancer Center and State Key Laboratory of Biotherapy, West China Hospital, West China Medical School, Sichuan University, Chengdu 610041, P.R. China;; 2 Department of Oncology, Sichuan Academy of Medical Sciences, Sichuan Provincial People’s Hospital, Chengdu, Sichuan 610041, P.R. China

**Keywords:** radiation pneumonitis, quercetin, oxidative stress, liposome

## Abstract

In the present study, the hypothesis that quercetin liposomes are able to effectively protect against radiation-induced pulmonary injury in a murine model was tested. C57BL/6J mice receiving whole-thorax radiotherapy (16 Gy) were randomly divided into three groups: control, radiation therapy plus saline (RT+NS) and RT plus quercetin (RT+QU). At 1, 4, 8 and 24 weeks post-irradiation, lung injury was assessed by measuring oxidative damage and the extent of acute pneumonitis and late fibrosis. In the lung tissues from the RT+NS group, the malondialdehyde (MDA) levels were significantly elevated and superoxide dismutase (SOD) and glutathione peroxidase (GSH-PX) activities were significantly reduced; the total cell counts and inflammatory cell proportions in the bronchoalveolar lavage fluid (BALF), plasma tumor necrosis factor (TNF)-α and transforming growth factor (TGF)-β1 concentrations and the hydroxyproline (HP) content were significantly increased. Quercetin liposome administration significantly reduced the MDA content and increased SOD and GSH-PX activities in the lung tissues, and reduced the total cell counts and inflammatory cell proportions in the BALF, plasma TNF-α and TGF-β1 concentrations and the HP content in the lung tissues. A histological examination revealed suppression of the inflammatory response and reduced TGF-β1 expression and fibrosis scores. Radiation-induced oxidative damage ranged from pneumonitis to lung fibrosis. Quercetin liposomes were shown to protect against radiation-induced acute pneumonitis and late fibrosis, potentially by reducing oxidative damage.

## Introduction

Radiation therapy (RT) is currently the cornerstone of the treatment of malignant thoracic diseases, including lung cancer, breast cancer, malignant lymphoma, esophageal cancer and thymoma. To ensure coverage of the tumor, the irradiation of normal tissues surrounding the tumor is unavoidable and may result in symptomatic injury (1). Radiation-induced pulmonary injuries (RIPIs), including radiation-induced pneumonitis and lung fibrosis, limit the therapeutic ratios of tumor treatment and reduce the quality of life in long-term survivors (1). Thus, effective prevention and control of RIPIs is extremely important in these patients.

The pathological process of RIPIs is complex, beginning with an acute inflammatory response that includes alveolar cell depletion and interstitial inflammation in the lung. Irreversible fibrosis, including fibroblast proliferation with collagen accumulation, occurs in the late stages of this process and eventually leads to the loss of normal lung structure (2). The biological effects of ionizing radiation begin with the direct generation of various reactive oxygen species (ROS), which cause oxidative damage to DNA, proteins and lipids, as well as the activation of transcription factors and signal transduction pathways (3). Oxidative damage to cellular components in the lung leads to cell damage and even cell death, and triggers inflammation that induces reparative processes and results in radiation-induced lung fibrosis (4). Thus, molecules with radical-scavenging properties show particular promise as radio-protectors (5). Animal studies have shown that antioxidant therapy reduces the extent of radiation-induced lung damage: hydrogen therapy has been shown to reduce cell damage, improve the viability of ionizing A549 cells and attenuate irradiation-induced damage by reducing oxidative stress (6); a superoxide dismutase (SOD) mimetic has been demonstrated to increase the tolerance of ionizing radiation in the lungs of rats (7); and amifostine was shown to reduce radiation-induced damage by scavenging oxygen and oxygen free radicals (8).

Quercetin, or 3,3’,4’,5,7-pentahydroxyflavone, is categorized as a flavonol, one of the six subclasses of flavonoid compounds (9). The protective effects of flavonoids in biological systems are ascribed to their capacity for transferring electrons to free radicals, activating antioxidant enzymes and inhibiting oxidative stress (10). Quercetin has a superior antioxidant activity due to the presence of the catechol group in the B ring and the OH group at position 3 on the AC ring. These structural features allow quercetin to donate hydrogen to scavenge free radicals and increase the stability of flavonoid radicals (11). Quercetin is known to possess marked antioxidative, anti-inflammatory and antifibrotic capacities. Animal experiments have demonstrated its ability to scavenge oxygen free radicals, inhibit lipid oxidation and affect the glutathione redox status (12,13). Quercetin has been shown to improve the inflammatory status by reducing tumor necrosis factor (TNF)-α and inducible nitric oxide synthase production in obese Zucker rats (14). *In vitro*, quercetin inhibits keloid fibroblast proliferation, collagen production and keloid contraction by suppressing transforming growth factor (TGF)-β/Smad signaling (15). *In vivo*, quercetin has been shown to improve liver histology and reduce collagen content in rats with carbon tetrachloride-induced cirrhosis (16). We thus hypothesized that quercetin would be an ideal candidate for the amelioration of RIPIs.

At present, the routine treatment for acute radiation pneumonitis includes a combined regime of adrenal cortex hormones and antibiotics, but this treatment does not effectively prevent or cure radiation pneumonitis or fibrosis. The present study aimed to investigate the effect and potential mechanism of the action of quercetin liposomes on RIPIs in a murine model.

## Materials and methods

### Quercetin liposome preparation

Since quercetin naturally has poor water solubility, laboratory-prepared quercetin liposomes characterized by improved solubility and increased *in vivo* absorbability (State Key Laboratory of Biotherapy, West China Hospital, Sichuan University, Chengdu, Sichuan, China) were used.

The quercetin liposomes were prepared as described previously (17). Briefly, mixtures of lecithin/cholesterol/PEG 4000/quercetin in 13:4:1:6 weight ratios were dissolved in chloroform/methanol (3:1, v/v) and evaporated until dry under reduced pressure in a rotary evaporator. The dried lipid films were sonicated in 5% glucose solution in a homothermal container. The final products were concentrated, lyophilized under vacuum for 5 h and stored at −20°C. This end-product has good solubility and may be used directly or dissolved in saline intraperitoneally.

### Animal model and experimental protocol

All animal procedures were approved by the Laboratory Animal Care Committee of Sichuan Province. Female C57BL mice (Experimental Animal Center of Sichuan University, Chengdu, Sichuan, China) aged 6–8 weeks, with approximate body weights of 18–20 g, were used in this study.

A total of 69 mice were randomly divided into three groups: a control group; an RT plus saline (RT+NS) group that received intraperitoneal injections of 200 *μ*l saline 2 h prior to irradiation and on days 1–28 subsequent to RT; and an RT plus quercetin liposome (RT+QU) group that received intraperitoneal injections of 5 mg/kg quercetin liposome, based on a previous study (17), 2 h prior to irradiation and on days 1–28 subsequent to RT.

For the thoracic irradiation, the mice were anesthetized by the intraperitoneal administration of 10 ml/kg 3.5% chloral hydrate. A single dose of cobalt-60 γ radiation (GWXJ80; Nuclear Power Institute of China, Chengdu, China) was administered to the entire thorax (0.8953 Gy/min; source-skin distance, 80 cm) of each mouse. Organs above and below the thorax were shielded.

At 1, 4, 8 and 24 weeks post-RT, four or five mice in each group were sacrificed. Peripheral blood samples and bronchoalveolar lavage fluid (BALF) were obtained, the left lung was fixed in 4% paraformaldehyde and the right lung was cryopreserved at −80°C.

### Malondialdehyde (MDA) content and SOD and glutathione peroxidase (GSH-PX) activities

Tissue from one lobe of each right lung was homogenized in 5% phosphate-buffered saline. The homogenate was then centrifuged at 800 × g for 10 min and the clear upper supernatant fluid was used. The MDA content and the SOD and GSH-PX activities in the lung were measured using respective kits (Nanjing Jiancheng Bioengineering Institute, Nanjing, China) according to the manufacturer’s instructions.

### BALF analysis

At 1, 4, 8 and 24 weeks post-irradiation, the mice were sacrificed, an open tracheotomy was performed and a small plastic tube was inserted into the trachea. BALF was extracted three times with 2 ml physiological saline. The BALF was centrifuged (400 × g, 15 min) and the cell pellet was suspended in 1 ml modified Hank’s balanced salt solution. Total nucleated and differential cell counts were performed on cellular monolayers prepared by cytocentrifugation at 800 rpm for 10 min, followed by hematoxylin and eosin (HE) staining. The percentages of inflammatory cell types (including neutrophils and lymphocytes) that were present were assessed by differential counts of 400 cells.

### TNF-α and TGF-β1 concentrations in plasma

The TGF-β1 and TNF-α contents of the plasma were measured by sandwich enzyme-linked immunosorbent assays (ABC-ELISA, R&D Systems, Minneapolis, MN, USA), according to the manufacturer’s instructions.

### Hydroxyproline (HP) assay

Collagen deposition was estimated by determining the total HP content of one lobe of each right lung using alkaline hydrolysis (Nanjing Jiancheng Bioengineering Institute). Briefly, the lung tissue in the test tube was weighed, 1 ml hydrolyzate was added and hydrolysis was performed in a boiling water bath for 20 min to regulate the pH (pH 6.0–6.8). The hydrolyzate was diluted by adding activated carbon, then the contents of the tube were mixed thoroughly and centrifuged at 3,500 rpm for 10 min and 1 ml supernatant was tested. Once the reagents had been added to the reaction mixture, the supernatant absorbance was measured at 550 nm.

### Histology and immunocytochemistry

The left lungs were fixed by an intratracheal instillation of 4% paraformaldehyde, allowed to cure overnight, embedded in paraffin and cut into 5-*μ*m thick sections. Certain sections were stained with HE and Masson’s trichrome for the determination of collagen content. Pulmonary fibrosis was scored using the scale developed by Ashcroft *et al* (18). Briefly, entire fields of 15 sections were scanned and each field was graded visually on a scale ranging from 0 (normal) to 8 (total fibrotic obliteration of the field). The mean of the scores obtained for all fields was used as the visual fibrosis score. The remaining sections were immunocytochemically stained with anti-TGF-β1 antibody (Santa Cruz Biotechnology, Inc., Santa Cruz, CA, USA) to detect active TGF-β1 expression. Five fields were randomly selected for each mouse and three mice from each group were examined; thus, a total of 15 sections were analyzed for each group. The number of cells showing active TGF-β1 expression within each field was counted under a light microscope at ×400 magnification (CX41RF; Olympus; Tokyo, Japan).

### Statistical analysis

Data are presented as the mean ± standard deviation. The statistical analysis was performed by a one-way analysis of variance, followed by Dunnet’s t-test. P<0.05 was considered to indicate a statistically significant difference.

## Results

### MDA content and SOD and GSH-PX activity in lung tissue

**Figure 1.**
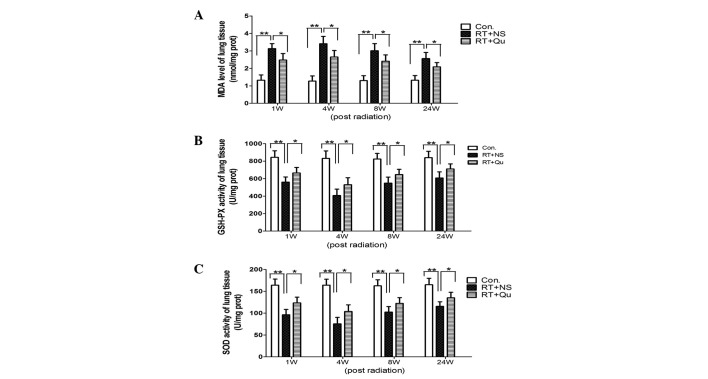
Measurement of (A) malondialdehyde (MDA) content and (B) glutathione peroxidase (GSH-PX) and (C) superoxide dismutase (SOD) activities in lung homogenates from experimental groups at 1, 4, 8 and 24 weeks post-irradiation. Values are expressed as the mean ± standard deviation (SD). ^**^P<0.01, ^*^P<0.05. Con, control; RT, radiotherapy; NS, saline; Qu, quercetin liposome.

From 1 to 24 weeks post-RT, the MDA content of the lung tissues increased significantly (all P<0.01 vs. control group; Fig. 1). Quercetin liposome administration significantly reduced the MDA content (all P<0.05 vs. RT+NS group).

From 1 to 24 weeks post-RT, the SOD and GSH-PX activities in the lung tissue significantly decreased (all P<0.01 vs. control group; Fig. 1). Quercetin liposome administration significantly increased the SOD and GSH-PX activities (all P<0.05 vs. RT+NS group).

### Total cell counts and proportions of inflammatory cells in BALF

**Figure 2.**
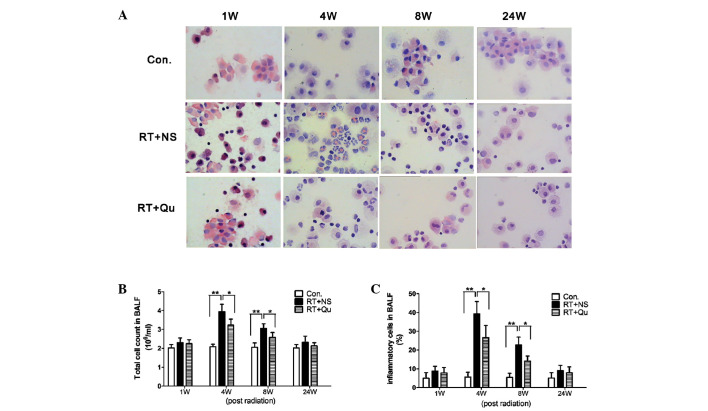
(A) Representative image of hematoxylin and eosin (HE)-stained cells in bronchoalveolar lavage fluid (BALF). Epithelial cells and macrophages were observed in samples from the control (CON) group. Significant increases in neutrophils showing nuclear lobulation and deeply stained small lymphocytes were observed in samples from the radiotherapy plus saline (RT+NS) group. The numbers of neutrophils and lymphocytes were noticeably reduced in samples from the radiotherapy plus quercetin liposome (RT+QU) group. Magnification, ×400. (B) Total cell counts and (C) percentages of inflammatory cells in BALF. ^**^P<0.01, ^*^P<0.05.

Epithelial cells and macrophages were the main cell types identified in the BALF from rats in the control group and the presence of lymphocytes were rare (Fig. 2). At 4 and 8 weeks post-RT, the total cell counts of the BALF and the percentages of inflammatory cells were increased significantly (all P<0.01 vs. control group). In the RT+QU group, the total cell counts of the BALF and the percentages of inflammatory cells were significantly reduced (all P<0.05 vs. RT+NS group) at 4 and 8 weeks post-RT.

### TNF-α and TGF-β1 concentrations in plasma

**Figure 3.**
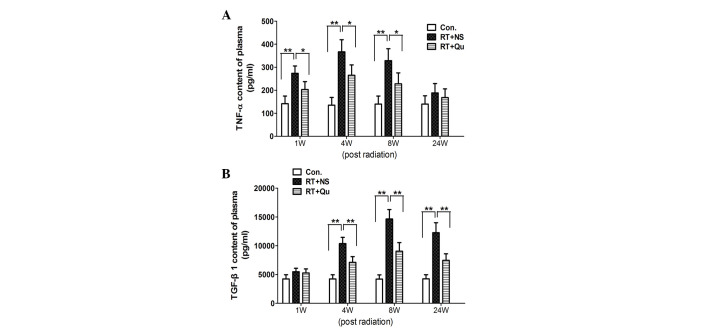
Dynamic changes in (A) tumor necrosis factor (TNF)-α and (B) transforming growth factor (TGF)-β1 concentrations in plasma from 1–24 weeks post-irradiation in the experimental groups. n=4–5 mice per group at 1, 4, 8 and 24 weeks. ^**^P<0.01, ^*^P<0.05. Con, control; RT, radiotherapy; NS, saline; Qu, quercetin liposome.

In the control group, the TNF-α concentration in plasma was 135.1±33.6 pg/ml (Fig. 3). At 1, 4 and 8 weeks post-RT, the TNF-α concentrations increased significantly, resulting in concentrations of 273.4±32.2, 367.0±52.5 and 328.8±51.7 pg/ml, respectively (all P<0.01 vs. control group). In the RT+QU group, the TNF-α concentrations declined significantly, with results of 203.1±34.2, 264.7±45.4 and 228.0±47.3 pg/ml, respectively (all P<0.05 vs. RT+NS group).

In the control group, the TGF-β1 concentration in plasma was 4,207.2±732.1 pg/ml (Fig. 3). At 4, 8 and 24 weeks post-RT, the TGF-β1 concentrations increased significantly to 10,373.2±1,084.8, 14,650.6±1,632.6 and 12,262.5±1,740.7 pg/ml, respectively (all P<0.01 vs. control group). In the RT+QU group, the TGF-β1 concentrations declined significantly to 7,100.8±1,009.4, 9,056.6±1,484.5 and 7,466.8±1,138.5 pg/ml, respectively (all P<0.01 vs. RT+NS group).

### Histological changes in lung tissue

**Figure 4.**
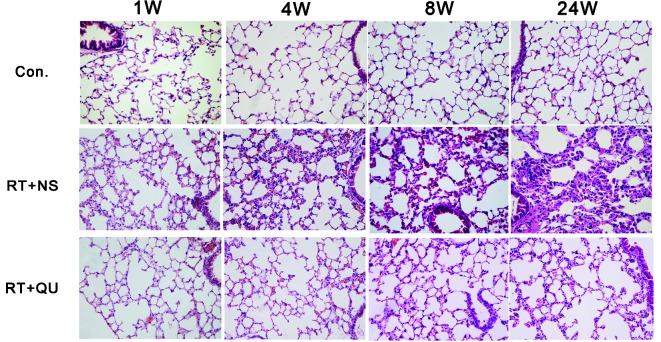
Photomicrographs of hematoxylin and eosin (HE)-stained lung sections from the control (CON), radiotherapy plus saline (RT+NS) and radiotherapy plus quercetin liposome (RT+QU) groups at various timepoints after irradiation. Magnification, ×400.

**Figure 5.**
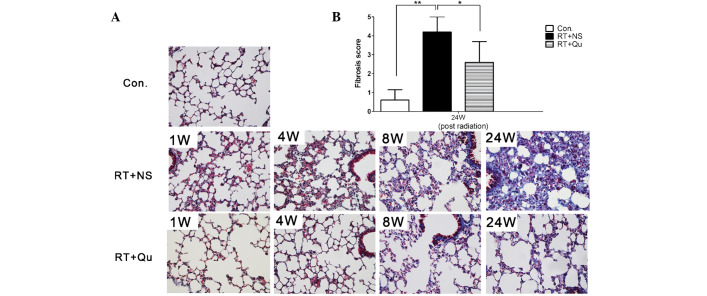
(A) Masson’s trichrome staining of lung tissue sections, with collagen fibers dyed blue. Magnification, ×400. (B) Lung fibrosis scores at 24 weeks after irradiation. Con, control; RT, radiotherapy; NS, saline; Qu, quercetin liposome.

**Figure 6.**
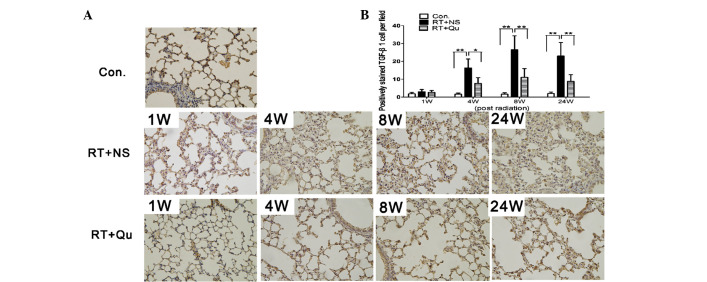
(A) Immunocytochemical staining with anti-transforming growth factor (TGF)-β1, with the cytoplasm of cells expressing TGF-β1 stained brown. Magnification, ×400 (B) Number of stained cells was counted within each field. Values are expressed as the mean ± standard deviation (SD). ^**^P<0.01, ^*^P<0.05. Con, control; RT, radiotherapy; NS, saline; Qu, quercetin liposome.

Subsequent to irradiation, two pathological phases of RIPIs were observed; an initial phase of acute and subacute pneumonitis (1–8 weeks), followed by late fibrosis (24 weeks; Figs. 4 and 5). In the pneumonitis phase, numerous inflammatory cells infiltrated the alveoli and alveolar walls and the lung interstitium was thickened, although minimal fibrosis was present on the alveolar walls, which were stained with Masson’s trichrome. In the late fibrosis stage, lung interstitium thickening and inflammatory cell infiltration were observed and the alveolar structure became disordered and collapsed. Notably, Masson’s trichrome staining revealed diffuse fibrous changes in the alveolar walls. The lung fibrosis score at 24 weeks post-irradiation was significantly higher (4.2±0.8) compared with the control group (0.6±0.55; P<0.01). However, the damage was clearly minor in the RT+QU group, with a lung fibrosis score of 2.6±1.1 at 24 weeks post-irradiation (P<0.05). The cells with active TGF-β1 expression infiltrated the lung tissue between 4 and 24 weeks post-irradiation (Fig. 6), although the degree of infiltration was significantly lower in the RT+QU group compared with the RT+NS group (all P<0.05 vs. RT+NS group).

### HP content in the lung tissue

**Figure 7.**
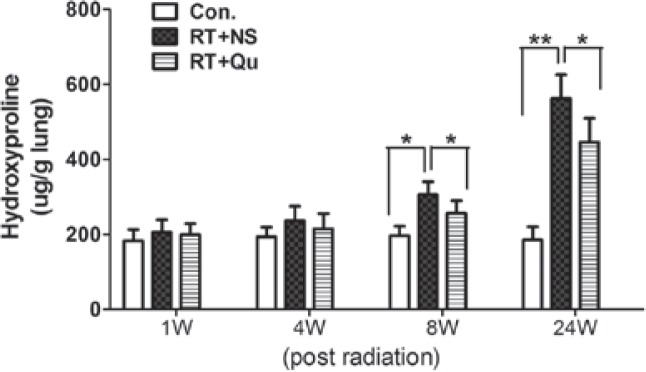
Collagen deposition was estimated by determining the hydroxyproline (HP) content of lung tissue in 4–5 mice per group at 1, 4, 8 and 24 weeks post-irradiation. ^**^P<0.01, ^*^P<0.05. Con, control; RT, radiotherapy; NS, saline; Qu, quercetin liposome.

Fibrosis is characterized by collagen deposition, and the HP content in the lung tissues reflects the proportion of tissue with collagen fibers. The lung tissues in the control group contained 185.5±34.4 *μ*g HP/g lung (Fig. 7). The HP content began to increase significantly in the first 8 weeks post-irradiation and peaked at 24 weeks (562.7±63.2 *μ*g/g wet tissue; P<0.01). Quercetin liposome administration noticeably reduced the HP content of the lung tissue (446.0±64.1 *μ*g/g lung at 24 weeks; P<0.05).

## Discussion

The present findings of marked increases in MDA content and reductions in SOD and GSH-PX activities between 1 and 24 weeks after whole-lung irradiation demonstrated oxidative stress sustained from radiation-induced pneumonitis and lung fibrosis. Ionizing radiation causes DNA damage through direct and indirect mechanisms (19); sensitive molecules in cells are directly damaged and interactions between radiation and water molecules in cells lead to the production of ROS, including superoxide anion radicals, hydrogen peroxide and hydroxyl radicals. Hydroxyl radicals are responsible for an estimated 60–70% of all ionizing radiation-induced cell damage (3,19). The radiation-induced burst of ROS generation is transient, but radiation also damages critical biomolecules governing the metabolic production of ROS, including mitochondria and oxidoreductase enzymes. Oxidative stress also contributes to the biological effects of ionizing radiation long after exposure (20). Leach *et al* (21) reported that the transient generation of ROS occurs within minutes of cell exposure to ionizing radiation, damaging mitochondrial permeability and resulting in the constant enhancement of ROS generation. Previous studies have shown that oxidative damage is increased and antioxidative capacities are decreased in radiation-induced lung injury (6,22). The effective protection of antioxidants have also been shown to indirectly reflect the potential causative role of oxidative stress in the development of RIPIs (7).

The antioxidant activities of quercetin are attributed to numerous factors, including free radical scavenging, protection against lipid oxidation (23), up-regulation of anti-oxidant enzymes and ROS trapping by direct hydrogen ion donation (12). However, quercetin administration has been hampered by its extreme water insolubility. The encapsulation of quercetin in liposomes improves its water solubility, prolonging circulation times in the blood and accumulation times in the lung (17). Significantly, the use of liposomal quercetin was shown to reduce the injection dose compared with free quercetin (17). Hence, in the present study, intraperitoneal injections of quercetin liposome were administered prior to and following RT. A lower MDA content and higher SOD and GSH-PX activities were observed in the lung tissue in the RT+QU group compared with the RT+NS group, demonstrating that quercetin inhibited pulmonary oxidative damage.

Inflammation may be central in the initiation and establishment of RIPIs (2). Changes in cell populations in the BALF have often been considered to reflect inflammatory changes in the lung (24). As a pro-inflammatory cytokine, TNF-α is likely to be involved in the early phase of RIPIs. Hence, the proportions of inflammatory cells in the BALF and TNF-α concentrations in the plasma were measured to estimate the extent of the inflammatory response. It was observed that the quercetin liposome significantly decreased the total cell counts and the proportion of inflammatory cells in the BALF, and also reduced plasma TNF-α concentrations. A histological examination revealed the suppression of the inflammatory response in the RT+QU group. The anti-inflammatory effects of quercetin may be attributed to the interplay between oxidative stress and inflammation (23). ROS are involved not only in the occurrence of oxidative stress, but also in the promotion of inflammatory processes. ROS are key mediators of inflammatory reactions in atherosclerosis (25). They are able to activate transcription factors such as nuclear factor (NF)-κB and activator protein-1, which induce the production of cytokines such as TNF-α (26). Consequently, ROS scavenging not only prevents oxidative stress, but also mitigates inflammation. Quercetin has been reported to inhibit TNF-α production and gene expression via NF-κB modulation (27). In animal models of allergic airway inflammation and asthma, quercetin has been demonstrated to reduce inflammatory cell infiltration and inflammatory cytokine production (28).

Tissue fibrosis is the excessive accumulation of collagen. TGF-β1 is a key cytokine in the fibrotic process that activates myofibroblast progenitors and upregulates collagen protein synthesis (2). Hence, in the present study, the plasma TGF-β1 concentrations and HP content in the lung tissue were measured, and Masson’s trichrome staining was used to estimate the extent of lung fibrosis. It was observed that quercetin liposomes significantly reduced the plasma TGF-β1 concentrations and HP content in the lung tissue. A histological examination revealed the suppression of TGF-β1 expression and collagen deposition in the lung. The lung fibrosis scores were significantly lower in the RT+QU group compared with the RT+NS group. The mechanism of quercetin’s antifibrotic effects may also be associated in part with the reduction of oxidative stress (29). Oxidative stress is postulated to play an important role in a wide range of fibrotic diseases, including atherosclerosis, cardiac fibrosis and idiopathic lung fibrosis (30). ROS and lipid peroxidation products stimulate fibrogenic cytokines that act as chemoattractants, mitogens and differentiating agents for smooth muscle cells (25,31). TGF-β isoforms are secreted in a latent complex, and the release of TGF-β from this complex is called activation. The ROS-mediated oxidation of a methionine residue in the latent complex releases TGF-β from extracellular reservoirs (32). *In vitro*, quercetin has been shown to suppress TGF-β-induced collagen production in normal human lung fibroblasts (33). In biliary-obstructed rats, quercetin has been shown to maintain an antioxidant defense and reduce oxidative damage, ameliorating liver fibrosis (29).

In conclusion, oxidative stress in the lung leads to radiation-induced pneumonitis and lung fibrosis. The present study demonstrated that quercetin liposomes inhibit pulmonary oxidative stress, alleviating radiation-induced acute pneumonitis and late fibrosis. Thus, quercetin effectively protected lung tissue against RIPIs.
